# Fracture strength test of digitally produced ceramic-filled and unfilled dental resin restorations via 3d printing: An *in vitro* study

**DOI:** 10.4317/jced.60173

**Published:** 2023-02-01

**Authors:** Ansgar-Christian Schulz, Ahmed Othman, Dragan-Alexander Ströbele, Juliane Wagner, Richard Mosch, Constantin von See

**Affiliations:** 1 Research Center for Digital Technologies in Dentistry and CAD/CAM, Department of Dentistry, Faculty of Medicine and Dentistry, Danube Private University, 3500 Krems, Austria. Steiner Landstraße 124, 3500 Krems, Austria; 2 Assistant Professor Research Center for Digital Technologies in Dentistry and CAD/CAM, Department of Dentistry, Faculty of Medicine and Dentistry, Danube Private University, 3500 Krems, Austria. Steiner Landstraße 124, 3500 Krems, Austria; 3 Research Center for Digital Technologies in Dentistry and CAD/CAM, Department of Dentistry, Faculty of Medicine and Dentistry, Danube Private University, 3500 Krems, Austria. Steiner Landstraße 124, 3500 Krems, Austria; 4 Department of Oral & Maxillofacial Surgery, University Hospital Schleswig-Holstein, Campus Kiel, Arnold-Heller-Straße 3, 24105 Kiel, Germany; 5 Research Center for Digital Technologies in Dentistry and CAD/CAM, Department of Dentistry, Faculty of Medicine and Dentistry, Danube Private University, 3500 Krems, Austria. Steiner Landstraße 124, 3500 Krems, Austria; 6 Director Research Center for Digital Technologies in Dentistry and CAD/CAM, Department of Dentistry, Faculty of Medicine and Dentistry, Danube Private University, 3500 Krems, Austria. Steiner Landstraße 124, 3500 Krems, Austria

## Abstract

**Background:**

Purpose of this study was to investigate the mechanical efficiency of 3D-printed permanent and provisional implant cemented fixed bridges produced via CAD/CAM technology using an interim and a permanent ceramic filled hybrid material.

**Material and Methods:**

Two groups with twenty specimens each were designed and 3D-printed via digital light processing technology (DLP). A fracture strength test was performed. Statistical analysis was performed (*p*>0.05) for impression distance and force.

**Results:**

For the fracture resistance and impression distance no significant difference (*p* = 0.643) were detected. The specimens of interim resin showed a mean value of 365.90 ± 86.67 N. Whereas specimens of permanent ceramic filled hybrid material showed a mean value of 363.45 ± 87.57 N.

**Conclusions:**

In this *in vitro* study 3D-printed ceramic filled hybrid material and interim resin based on methacrylic acid esters showed an acceptable resistance to bite forces with no differences in fracture mechanism.

** Key words:**CAD-CAM, dental resin, 3D printing.

## Introduction

A patient’s poor oral hygiene level or chronic medical problems can result in the loss of teeth. A reduced number of teeth worsens food intake due to deteriorated chewing performance, which can affect quality of life (1). As a result, the demand of fixed restorations including dental implants increases (2). Missing tooth without replacement ensures that the occurring chewing forces are poorer distributed. In addition, increased abrasion can lead to a reduction in the vertical dimension of occlusion (VDO) (3). Therefore, the selective requirements for a temporary or permanent restorative material to withstand the masticatory forces maintain occlusion stability, aesthetics and phonetic restoration are to be considered mandatory (4-10).

The restorative material plays an important role in soft tissue management, occlusal stability and patient acceptance (11). Therefore, when selecting the material, it must be taken into account that ceramics mimic the optical properties of natural teeth and are more prone to fracture, especially when subjected to force (12,13).

All different interim or permanent materials have to withstand the chewing forces to a certain extent, but this differentiability leads to different chemical and mechanical properties of the materials and thus to different uses.

In general, there are temporary and permanent materials. Temporary materials can be divided into long-term materials and short-term materials. The chemical constituents are responsible for physical properties of restorative materials. This could be evaluated in material breaking strength when being tested using mechanical force (14).

The digital production of provisional or permanent restorations with CAD / CAM technology (computer-aided design and computer-aided manufacturing) is widely used and has advanced the development of dental restorations, particulary in the areas of speed and reproducibility (15-17). Some studies have already shown that the restorations produced by CAD / CAM systems offer a quality standard that corresponds to or even exceeds that of non-digital methods (18-20).

A subtractive or milling manufacturing of restorations is always accompanied by the problems of material waste and the risk of micro-cracks (21,22).

3D printing also known as additive manufacturing is another way of manufacturing dental restorations, especially for resin and with limits also for ceramics (17,23,24). Materials can be printed in an incremental vertical build-up with less material wastage and no force application (25). 3D printing has many other advantages including the ability to create structures in multiple materials or emitting less noise and heat (26,27). There are several prospects in additive manufacturing technology for dental ceramic crowns with different fracture strength (28-30).

Using either stereolithography (SLA) or digital light projection (DLP), 3D printers produce 3D objects by polymerizing liquid photopolymers with an ultraviolet (UV) laser or UV light-emitting diode (LED) (31). SLA printers use a laser point to draw precise patterns on the bottom of a material container, allowing the liquid light-curing resin to harden layer by layer (32). DLP printers which uses a light controlled by a digital mirror device (DMD), the DMD allows a fast process by curing an entire layer at a time (33,34).

A 3D printed resin represents as an enduring aesthetic restorative possibility for permanent or interim long-term implant cemented prosthetics, however, only limited data on fracture strength are available for recent developments (35).

Therefore, a systematic test of the mechanical behavior such as fracture strength for temporary or permanent materials via in-vitro tests is considered useful prior to clinical usage. This makes it possible to assess the behavior of 3D printed restorations in clinical situations.

The objective of the present *in vitro* study was to mechanically examine the fracture strength and impression distance of 3D printed permanent and provisional implant cemented fixed bridges produced via CAD/CAM technology based on methacrylic acid esters using interim resin (VarseoSmile Temp A2, BEGO, Bremen) and the permanent ceramic filled hybrid material resin (VarseoSmile Crown Plus A2, Bego, Bremen, Germany) to enable a safe and longterm usage of these prosthetic restorations in the future.

## Material and Methods

A power analysis was performed to determine the sample size using the G*Power software version 3.1.9.7 (Heinrich Heine Universität, Düsseldorf, Germany) (36). Meaningful values for power with 80% and an effect size 0.8 were chosen (37).

Two groups with twenty specimens each with the same digitally designed bridge were produced in ten different cycles via CAD/CAM technology. Accordingly, all specimens are identical designed using computer-aided dental system program (3Shape, Copenhagen, Denmark).

**Figure 1:**
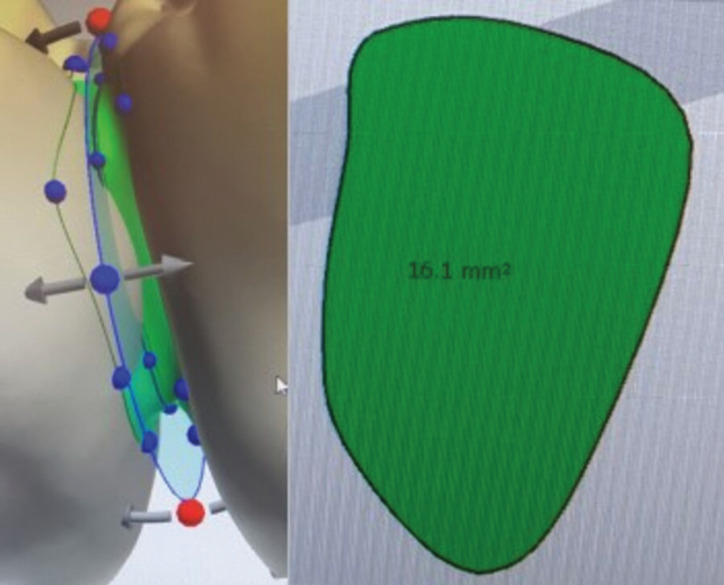
Cross sectional area of the connector 16,1 mm² showed in computer-aided dental system program (3Shape, Copenhagen, Denmark).

The design of the bridge restoration from first premolar to first molar was adopted due to the recommendation of the software 3Shape except some necessary modifications for the 3D printing process. The VST group is identified by provisional resin material VarseoSmile Temp A2 (REF 41022, LOT 600046, Bego, Bremen, Germany) while group VSCP with permanent resin material VarseoSmile Crown Plus A2 (REF 41108, LOT 600018, Bego, Bremen, Germany). The chemical composition of the group VST material, are esterification products of 4.4’-isopropylidenediphenol, ethoxylated and 2-methylprop-2-enoic acid and diphenyl (2,4,6-trimethylbenzoyl) phosphine oxide. The chemical composition of the group VSCP material, are esterification products of 4.4’-isopropylidenediphenol, ethoxylated and 2-methylprop-2-enoic acid. Silanized dental glass, methyl benzoylformate, diphenyl (2,4,6-trimethylbenzoyl) phosphine oxide. Total content of inorganic fillers (particle size 0.7 μm) is 30 – 50 % by mass. The samples were designed as the recommended minimal wall thickness of 1.5 mm and connectors cross-sectional areas of 16 mm2 for bridges in posterior teeth area for the VarseoSmile Temp material (Fig. 1).

Each cycle was 3D printed via digital light processing technology (DLP) using Varseo XS printer (Bego, Bremen, Germany). Five cycles for group VST and five cycles for group VSCP.

The post processing of all 3D printed specimens was performed as recommended by manufacturer’s instructions. First the specimens were cleaned for three minutes in an unheated ultrasonic bath, in a container of reusable ethanol with a concentration of 96 %. Second, two minutes in a container with fresh ethanol (96 %) in an unheated ultrasonic bath.

The specimens were withdrawn from the ethanol bath, remaining resin residues were removed with apply-tips (Hager & Werken, Duisburg, Germany) soaked in 96 % fresh ethanol. Then the specimens were dried with oil free compressed air, support structures were removed and fitting was checked. Post-curing process was undertaken using nitrogen gas (1.0 - 1.2 bar) and otoflash (Bego, Bremen, Germany) of 10 Hz (Hertz) with 1500 flashes were made each of two cycles, the model was turned after first cycle. After that the specimens were cemented on implants. Both groups were characterized by the manufactured method and material used.

Accordingly, in the present investigation, the adhesive luting system Variolink Esthetic (Ivoclar Vivadent, Schaan, Liechtenstein) was used for cementation. No treatment was provided before luting due to lack of improvement with any primer. The luting composite is approved for this type of restoration and is used clinically on a regular basis. The cementation is always dispensed from the automix syringe in the optimum ratio. The choice to use this luting material came about due to better reproducibility and the frequent clinical usage of the material.

**Figure 2:**
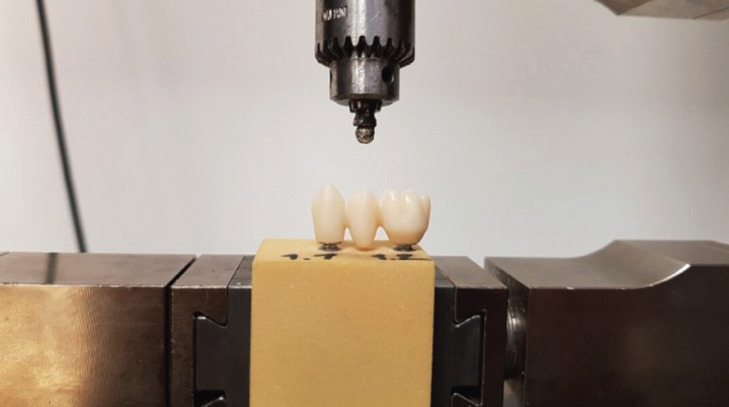
Bone block with implants and bridge positioned in Zwick/Roell universal test machine Z010.

All specimens were cemented on implants SICvantage max with diameter 4.2 mm and length 11.5 mm (REF 950184, LOT 619617, SIC, Basel, Switzerland) and a SICvantage max abutment red, straight, gingiva height 1.0 mm (REF 950641, LOT 1910021076, SIC, Basel, Switzerland) which were placed into artificial bone blocks (Sawbones, Vashon Island, Washington, USA) (Fig. 2).

The distance of the implants placed was from centre to centre 16.0 mm. The mechanical test was performed with Zwick/Roell universal testing machine Z010 (Zwick/Roell, Ulm, Germany).

Based on ISO standard 178, which standardizes a 3-point bending test, a fracture strength test was carried out on the real geometry of a bridge restoration until the material fatigued. A ball made of hardened metal (bearing steel: 1.3505/100Cr6/AISI 52100, E-modulus: 210 GPa) with a suitable size for a maxillary stamping cusp (diameter of 2.5 mm) with a preload force set to 10 N (Newton) were positioned at a distance of 0.8 mm. The testing speed was 60 mm/min. Hardened metal ball pressured each specimen at the middle of the pontic in the central forsa of the second premolar of each fixed bridge in a parallel direction of force to the abutment (4).

The bridges were loaded until the maximum force was reached. All results were recorded in Newton and divided into F (Force) maximum and F break with the maximum displacement value for F maximum and F break for each specimen.

Statistical analysis was performed for the force and displacement distance (*P* values less than 0.05 were regarded statistically significant).

## Results

**Figure 3:**
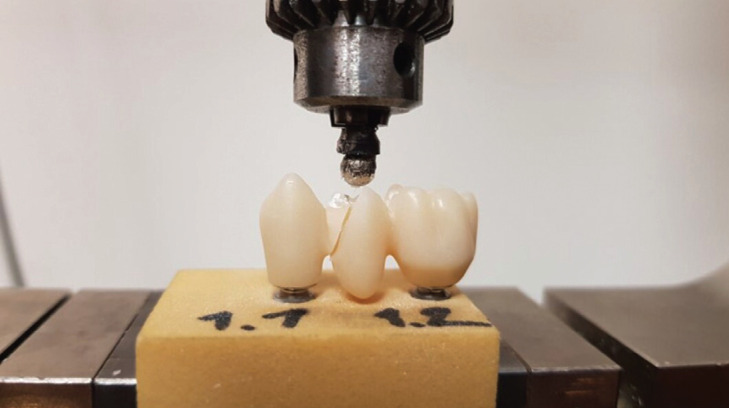
Bridge with fracture line after the breaking point was reached and the metal ball back in beginning position.

The fracture surface of the specimens in both groups was nearby to a connector area in the pontic of the bridge (Fig. 3).

All specimens broke in two to four parts. First the lingual cusp of the second premolar broke. Sometimes this led to a reduction in the measured force for a short time. Then there was a steady increase in the force measured until the specimen suddenly broke.

The main fracture line draw crosswise to the course of the bridge between the first and the second premolar, involving the pontic partly with participation of the first premolar but never with participation of first molar. The crack was in the full length of the connector and the crack area was over 16,1 mm2. Using the shapiro-wilk normality test showed for group VST *p* = 0.698 and for group VSCP *p* = 0.589. The data showed a normal distribution of the specimens. Shapiro-wilk normality test *p*>0.05. Using a paired t-test to compare group VST and group VSCP showed *p* = 0.643, so there is no statistically significant difference. The material in group VST showed a maximal deformation in three specimens with approximately 1.9mm when applying 348 N, 365 N or 192 N. The maximum deformation was recorded at 2.2 mm when applying 373 N to group VSCP. In addition, the force break was analyzed (Table 1).

**Table 1:**
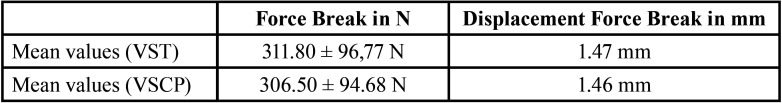
Mean Values of VarseoSmile Temp (Group VST) and VarseoSmile Crown Plus (Group VSCP).

**Figure 4:**
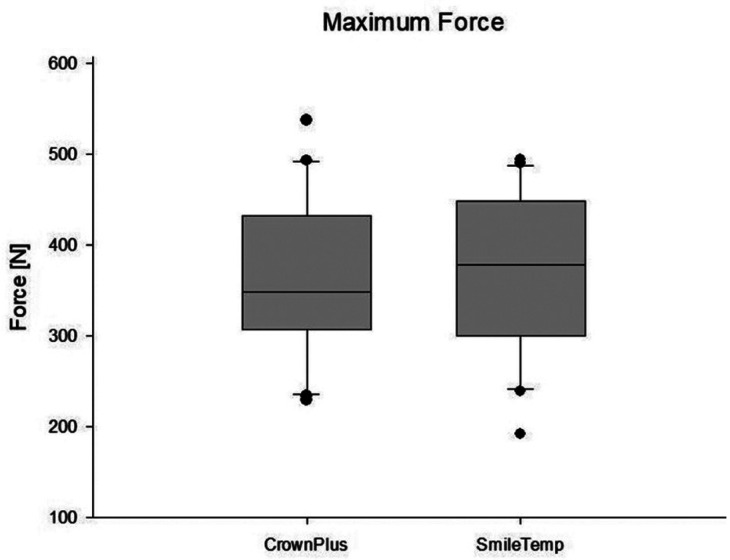
Comparative graph illustrating the forces loaded on both groups.

Moreover, the maximum force to specimens was investigated (Fig. 4). The specimens of group VST showed a mean value of 365.90 ± 86.67 N, median of 377.7 N, 1st quartile of 312.3 N to 3rd quartile of 432.9 N and a minimum of 192.2 N and a maximum of 494.2 N. The values are within the expected range (38).

The specimens of group VSCP showed a mean value of 363.45 ± 87.57 N, median of 347.9 N, 1st quartile of 310.6 N to 3rd quartile of 427.7 N and a minimum of 229.5 N and a maximum of 537.3 N.

The main aim of the present investigation was to find the material-specific properties in-vitro, not to imitate the natural bite forces or chewing movements. A systematic in-vitro testing is essential for fundamental understanding of the materials used.

All group values are illustrated by force-displacement graph using SigmaPlot software version 13.0 for windows (Sytstat Software Inc, San José, USA).

## Discussion

The aim of this study was to analyze the influence of ceramic particles in additively manufactured resin restorations with regard to fracture strength. Especially interesting of this investigation was that the material of group VST which is a long-term provisional and indicated for crown and bridges (maximum seven units, maximum pontic width one molar), inlays, onlays and veneers and it was compared with the resin material of group VSCP for permanent prosthetics. The use of a permanent luting material serves to improve comparability in both groups.

Both materials are made of the same resin. But the chemical difference between the materials is that in group VSCP, the polymerized framework of the resin is ceramic-filled.

The results of the present investigation have shown that the mean values of 3D printed materials based on methacrylic acid esters (group VST) and a ceramic filled hybrid material based on metharcrylic acid esters (group VSCP) regarding the fracture strength were comparable. There was no statistically significant difference in the forces of either material at 80 % power and 0.8 effective size. In a different study design with a different power, different effective size or different number of samples, deviating results are possible.

In general, the influence of the cementation system might have led to higher values for the provisional material.

In 2019, a study was published by Holmer *et al*., who used the Variolink Esthetic to bond two printed dental resin materials for shear bond testing, showed the positive effect of cementation with a resin cement for VarseoSmile Temp (39). The Variolink Esthetic DC is characterized by high compressive and flexural strength. It has a wide range of dental indications, including the bonding of printed dental acrylics. In addition, Variolink Esthetic DC is a suiTable luting cement for use with a significantly higher bond strength to VarseoSmile Temp than Fuji Cem2 (39). However, the investigation examined the materials at one particular timepoint not taking material aging in account. Provisional restorations have to fulfil modern aesthetic demands, biocompatibility, maintenance of abutment alignment and mechanical functional resistance (7). Besides, natural teeth, like dental materials, are exposed to physiological and pathological abrasion (40). Even crowns and bridges made out of ceramics may lose their occlusal height due to abrasion, but otherwise causing the main occlusal loss of hard tooth tissue in natural anterior dentition (41). Moreover, regarding provisional materials it can be assumed that the inferior mechanical properties, cause a worse abrasion resistance that the natural dentition or even ceramic restorations (42). Hence, a main advantage of dental materials containing ceramic components like VarseoSmile Crown Plus could be the longterm behaviour especially for colour stability and wear resistance. Other studies could already show that provisional composite restorations show a statistically significant reduction of the occlusal plane and also of the colour stability (40). Further studies are needed to compare the properties of such materials at a later point of their lifecycle.

It is also questionable whether the post-curing process recommended by the manufacturer, which is the same for both materials, represents a disadvantage for a ceramic-filled material. The ceramic part may scatter the light and lead to poorer penetration of the light, which leads to a different degree of polymerisation. Also, the photo-polymerization device has an influence of the physical properties of materials (43). Adjusting the post-cure process with elevated temperature could make the polymerization more uniform and improve the physical properties. Another study has shown that wash time and wash solution may influence the mechanical properties of 3D-printed dental resins (44). Even though the median and mean of group VST was more than the minimum and maximum force resistance of group VSCP. The proportion of ceramic in the material of group VSCP did not lead to any statistically noticeable deterioration of fracture resistance in this *in vitro* study but could have led to higher minimum and maximum values.

But other investigations have shown that the proportion of ceramics lead to an improvement of other properties. A ceramic-filled hybrid material can increase the abrasion resistance (45), the surface resistance against roughness and loss of mass (46). Also, ceramic-filled hybrid material is competitive in long-term cementation stability, decementation behaviour and marginal gap formation (47). Additionally, it is possible that the classical disadvantage of a resin material, like the water absorption (48,49), which can lead to a decementation or discolouration (40,50), could be decreased by adding ceramic particles. Whether the proportion of ceramic in a resin framework can lead to a higher elasticity, would have to be examined more closely in further test.

A first indication of improved elasticity is due to the fact, that the distances the hardened metal ball was able to cover until the material breakage, are increased. The maximum value for group VSCP is 2.2 mm and for group VST 1.9 mm. In group VSCP, the distance to the break was increased by up to 15.8 %. These results are in line with investigations by Bona *et al*. for another polymer-infiltrated ceramic network material (51). Furthermore, clinical circumstances cannot be fully simulated in-vitro with a standardized test. However, it is possible to find material-specific properties in-vitro. The use of artificial sawbone blocks led to reproducible results. Sawbones provide an accurate reproduction of the biomechanical features of human bone when subjected to variable loads (52,53).

Systematic *in vitro* tests are important for a fundamental understanding of the materials used. A proven technique is the fracture strength, with which the mechanical test of the samples can easily be assessed.

In this investigation it was shown, that the ceramic filled resin material have comparable fracture resistance and flexibility properties while possessing lower deviation within the methacrylic resin material without ceramic.

## Conclusions

Within the limitations of this *in vitro* study and based on the results obtained, it can be concluded that a 3D-printed ceramic-filled hybrid material based on methacrylic esters can exhibit comparable resistance to masticatory forces as a 3D-printed methacrylic ester resin.
